# Integrating health literacy, health promotion, and digital health in tuberculosis prevention: An ecosystemic competency framework and CIMO-based systematic review

**DOI:** 10.14202/vetworld.2026.1888-1899

**Published:** 2026-05-10

**Authors:** Sri Sugiarsi, Endang Sutisna Sulaeman, Sapja Anantanyu, Anik Lestari

**Affiliations:** 1 Doctoral Program of Development Extension and Community Empowerment, Universitas Sebelas Maret, Surakarta, Indonesia; 2 Master’s Program in Public Health, Graduate School, Universitas Sebelas Maret, Surakarta, Indonesia; 3 Faculty of Medicine, Universitas Sebelas Maret, Surakarta, Indonesia

**Keywords:** behavioral determinants, digital health, ecosystemic framework, health literacy, health promotion, systematic review, tuberculosis prevention, public health interventions

## Abstract

**Background and Aim:**

Tuberculosis (TB) continues to pose a major global public health burden, particularly in low- and middle-income countries. Although biomedical interventions have advanced, prevention remains strongly influenced by behavioral, sociocultural, and systemic determinants. Health literacy, health promotion, and digital health innovations have demonstrated potential in improving TB-related outcomes; however, their integration within a unified theoretical framework remains limited. This study aimed to synthesize evidence on TB prevention strategies by integrating health literacy, health promotion, and digital health using the Ecosystemic Health Promotion Competency Framework (EHPCF) and the Context–Intervention–Mechanism–Outcome (CIMO) approach.

**Materials and Methods:**

A systematic review was conducted following the Preferred Reporting Items for Systematic Reviews and Meta-Analysis (PRISMA) 2020 guidelines. Literature published between 2015 and 2025 was retrieved from the Scopus database using structured keywords related to TB prevention, health literacy, health promotion, and digital health. Of 417 identified records, 49 studies met the inclusion criteria after screening and eligibility assessment. Data extraction was performed using a structured matrix, and thematic synthesis was applied. Study quality was assessed using the Mixed-Methods Appraisal Tool, while the CIMO framework guided analytical interpretation.

**Results:**

Health literacy consistently emerged as a significant predictor of preventive behaviors, with 34% of studies focusing on behavior relationships, 32% on determinants, and 28% on interventions. Community-based health promotion interventions improved self-efficacy, awareness, and health-seeking behavior, though long-term sustainability remained insufficiently evaluated. Digital health interventions, including mHealth and eHealth platforms, enhanced awareness and adherence but were constrained by inequitable access and digital divides. Structural and cultural determinants, such as poverty, stigma, and migration, significantly influenced TB prevention outcomes; however, these factors were rarely integrated into intervention models. The CIMO synthesis highlighted that context-specific mechanisms, including trust, social support, and stigma reduction, mediated improvements in preventive behaviors and adherence.

**Conclusion:**

This systematic review provides an integrative perspective linking health literacy, health promotion, digital health, and socio-structural determinants in TB prevention. The EHPCF offers a multidimensional competency-based framework, while the CIMO approach enhances understanding of intervention mechanisms across contexts. Future research should prioritize longitudinal and mixed-methods designs, address digital inequities, and validate integrative frameworks to support sustainable TB prevention strategies.

## INTRODUCTION

Tuberculosis (TB), remains a major health issue, with 10.6 million estimated new infections and 1.3 million estimated deaths in 2022, with most occurring in low- and middle-income countries worldwide [1] such as India [2–4]. However, despite advancements in biomedical interventions, which are undeniable in their effectiveness, particularly in improved diagnostic tests and treatments, “the control of TB remains, fundamentally, a matter of behavioral and systems change that is unaffected by pharmaceutical interventions” [5]. In this scenario, health literacy (HL) and health promotion (HP) are increasingly recognized as major determinants of health behavior in disease prevention activities [6, 7].

Over the years, theoretical models such as the Health Belief Model (HBM) and the Health Promotion Model (HPM) have been widely used to describe TB-related behaviors [8]. Evidence from studies in Nigeria, China, and Turkey, among others, shows that models can improve knowledge, attitudes, and health behaviors [9–11]. Yet they still have limited capability in explaining TB behaviors since factors like poverty, stigma, and migration are commonly overlooked and addressed either peripherally or overtly. Notably, however, community TB programs and health information technology innovations in mobile health and electronic health platforms have received attention and shown strong potential in various studies [12–14]. Yet, sustainability, scalability, and equal TB access remain largely unanswered in community TB programs and health information technology innovations in mobile health and electronic health platforms [15–17].

For example, past reviews adopted socioecological or pedagogical perspectives on HL and HP [18, 19]. Interestingly, though, none of the reviews presented an integrative framework that systematically linked HL with digital health and structural determinants, and simultaneously analyzed their effects on behavioral outcomes in TB prevention [20]. The review addresses emergent challenges (misinformation, digital divides amplified by the pandemic, fragile systems) in relation to End TB and Sustainable Development Goals 2030 [20].

In this light, the current article aims not only to make sense of the existing body of knowledge on TB prevention from 2015–2025 but also to advance the discourse by using the Ecosystemic Health Promotion Competency Framework (EHPCF). The latter framework defines HL from a competency construct encompassing the personal, sociocultural, systemic, and digital areas. Updated competencies and socioecological HL models include an explicit digital area as a core competency layer and an ecosystemic view that links individual HL to systemic and digital inequities in TB contexts. On a different note, the article also uses another framework, referred to as the Context–Intervention–Mechanism–Outcome (CIMO) approach, as a lens to argue that outcomes of interventions are generated by mechanisms specific to given contexts. Unlike Chauhan *et al*. [20], who focused on HL–adherence correlations or recent meta-analyses emphasizing adherence technology, this review integrates behavioral, digital, and socio-structural dimensions through EHPCF and CIMO. The two together not only fill a gap but also owe a debt to the lack of clarity in the discourse of the matter. This positions the paper as proposing a new integrative competency model for infectious disease prevention, filling a gap in TB-specific HL and HP theorizing.

This study aims to address these gaps by conducting a comprehensive systematic review of TB prevention strategies published between 2015 and 2025, with the objective of integrating HL, HP, and digital health within a unified theoretical and analytical framework. Specifically, the study seeks to synthesize empirical evidence to identify key determinants, intervention strategies, and outcomes associated with TB prevention, while simultaneously examining the contextual mechanisms that influence their effectiveness. To achieve this, the study applies the EHPCF to conceptualize HL as a multidimensional competency spanning personal, sociocultural, systemic, and digital domains.

In addition, the study uses the CIMO approach as an analytical lens to explore how interventions function within specific contexts and to uncover the mechanisms driving observed outcomes. By integrating these frameworks, the study aims to move beyond descriptive synthesis toward an explanatory and theory-driven understanding of TB prevention. Ultimately, this research proposes a novel, ecosystemic, competency-based model to guide the design, implementation, and evaluation of integrated TB prevention strategies, with particular relevance to high-burden, resource-constrained settings.

## MATERIALS AND METHODS

### Ethical approval

Ethical approval was not required for this study because it is a systematic review based exclusively on previously published data and does not involve direct interaction with human participants or the use of identifiable personal data. The study was conducted in accordance with internationally accepted ethical standards for research and reporting, including adherence to the Preferred Reporting Items for Systematic Reviews and Meta-Analyses (PRISMA) guidelines and principles of transparency, reproducibility, and scientific integrity.

All data included in this review were obtained from publicly accessible sources, and the original studies were assumed to have been conducted in compliance with their respective institutional and national ethical regulations. Proper citation and acknowledgment of all sources were maintained to ensure academic integrity and avoid plagiarism.

### Protocol registration

The review protocol was registered in PROSPERO (registration number CRD420261333758), ensuring methodological transparency and minimizing the risk of reporting bias.

### Study period and location

The search was conducted from January to March 2025 at Doctoral Program of Development Extension and Community Empowerment, Universitas Sebelas Maret, Surakarta, Indonesia.

### Search method

This systematic literature review is guided by the PRISMA 2020 guidelines proposed in previous studies [21, 22] (Supplementary File). In searching for literature, the database used is Scopus, a citation database selected for its comprehensive coverage of peer-reviewed journals and high-quality indexing [23].

The search produced a total of 417 records. After applying the time frame (years 2015–2025) with the final search completed on March, 31 2025, publication language (English only), as well as the exclusion of conference abstracts and non-indexed journals, the total records were reduced to 189 [23–25]. The titles, abstracts, and full-text articles were then screened for eligibility. Reference mining added one additional study.

The search strategy was designed using the Population, Intervention, Comparator, Outcome structure and combined controlled terms and free-text terms related to the following: (“*tuberculosis*” OR “*TB*” OR “*pulmonary tuberculosis*”) AND (“*health literacy*” OR “*health promotion*” OR “*health education*” OR “*digital health*” OR “*mobile health*” OR “*electronic health*” OR “*telehealth*”) AND (“*prevention*” OR “prevent*” OR “behavior*” OR “*adherence*” OR “*compliance*”). The search was conducted without language filters, focusing on human studies in adult populations. Search results were filtered using EndNote software (Clarivate Analytics, Philadelphia, PA, USA) to improve search management and avoid duplication while maintaining sensitivity.

### Review question and framework

Primary question: “What is the association between HL, HP interventions, and/or digital health innovations (as exposures/interventions) and preventive behaviors, adherence, or outcomes in TB prevention, and what contextual mechanisms drive these effects?”

This was formalized using a Population, Exposure, Comparator, Outcome framework, given the inclusion of both observational and interventional evidence. Population: individuals or communities at risk of or affected by TB, particularly in high-burden low- and middle-income countries and vulnerable populations such as migrants, the urban poor, and adolescents. Exposure/intervention: HL levels or tools, HP programs (e.g., peer education, community outreach), and digital health innovations (e.g., mobile health, short message service, electronic health platforms). Comparator: standard care, no intervention, or lower levels of exposure. Outcome: preventive behaviors (e.g., screening uptake, mask use), treatment adherence, health-seeking practices, awareness, self-efficacy, and reduced stigma or delay.

### Eligibility criteria

Inclusion criteria were studies that (i) focused on TB prevention in relation to HL, HP, or digital health; (ii) contained empirical findings in the form of quantitative, qualitative, or mixed-methods studies or theoretical frameworks; and (iii) were published in peer-reviewed journals indexed in Scopus within Q1 to Q4 categories.

Exclusion criteria were studies that (i) did not primarily focus on TB prevention; (ii) lacked primary empirical findings; and (iii) were abstracts, editorials, or commentaries.

### Data extraction and quality assessment

The full texts of 49 studies were evaluated. A structured extraction matrix was designed to extract key characteristics of the included studies, such as study design, population, setting, paradigm orientation, type of intervention, and outcomes. To improve coding objectivity, the Watase Uake system, an online collaborative coding platform, was used to enhance thematic consistency, as previously described [26]. Two reviewers independently screened and extracted data, and inter-rater agreement was assessed using the kappa statistic.

This system was used alongside the structured extraction matrix to categorize key elements before conducting thematic synthesis and CIMO analysis. Thematic synthesis followed the steps described by Thomas and Harden [27], including line-by-line coding, development of descriptive themes, and generation of analytical themes mapped to the CIMO framework.

The methodological quality of the included studies was assessed using the Mixed-Methods Appraisal Tool (MMAT), which provides a standardized approach for evaluating qualitative, quantitative, and mixed-methods studies [28, 29].

### Data synthesis and analytical lens

Thematic synthesis was employed to identify patterns and conceptual relationships across studies [26]. Interventions were grouped based on their theoretical frameworks, including HBM, HPM, theory of planned behavior, and socioecological perspectives, as well as intervention modalities such as literacy-based, technology-based, and community-based approaches. Outcomes were categorized into knowledge improvement, behavior change, and broader health outcomes.

In addition, the CIMO framework was applied as the primary analytical lens. This enabled systematic integration of context, intervention, mechanism, and outcome components. The framework facilitated understanding of how interventions operate within specific contexts to activate mechanisms such as self-efficacy and social support, ultimately leading to improved TB prevention outcomes.

The detailed mapping for the CIMO analysis is provided in the Supplementary File.

## RESULTS

### Study selection

**Figure 1:**
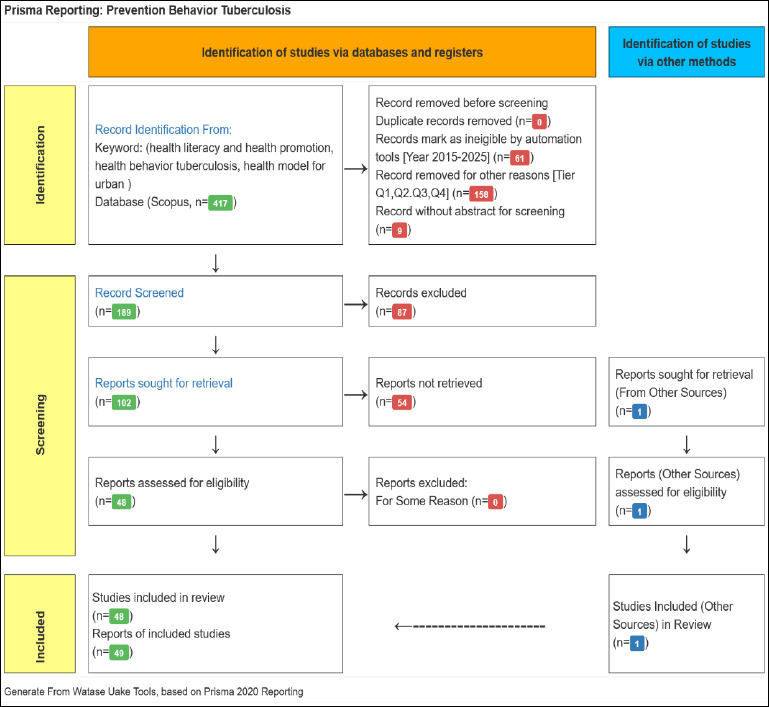
Preferred Reporting Items for Systematic Reviews and Meta-Analysis (PRISMA) flow diagram showing the identification, screening, eligibility, and inclusion process for studies on tuberculosis prevention, health literacy, and health promotion (2015–2025).

The search identified 417 records. After screening and eligibility assessment, 49 studies were included in the final synthesis (Figure 1).

### Keyword analysis

**Figure 2:**
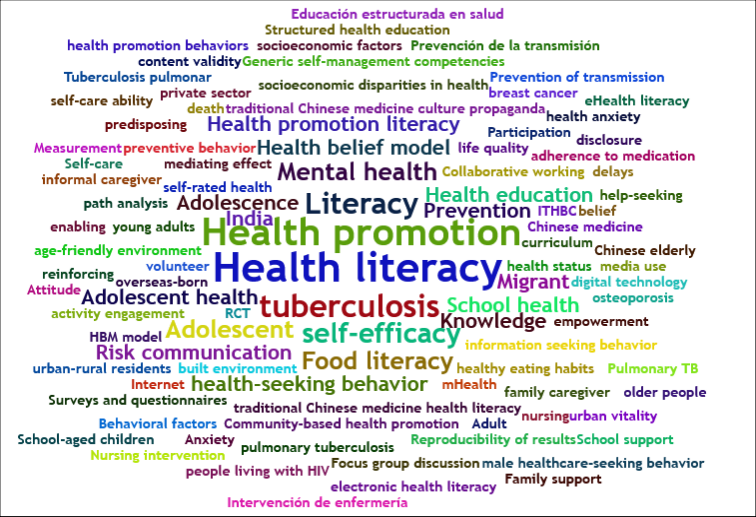
Keyword mapping of tuberculosis prevention research (2015–2025). The most frequent terms include health literacy, health promotion, and tuberculosis, with clusters related to self-efficacy, mobile health and electronic health, stigma, and school health.

Keyword mapping revealed HL, HP, and TB as the most frequent terms, with associated clusters including self-efficacy, stigma, mobile health and electronic health, and school health (Figure 2). These clusters indicate a growing integration of psychosocial and digital dimensions in TB prevention research.

### Publication trends

**Figure 3:**
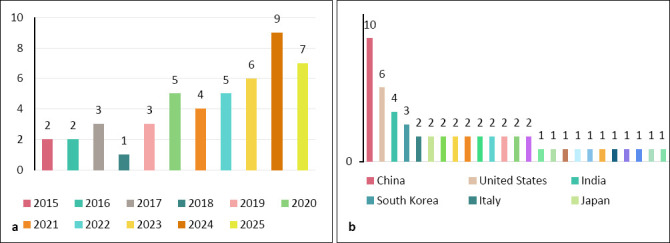
Annual and geographic publication trends. (a) Annual publication trends (2015–2025)(b) Geographic trends in tuberculosis prevention research (2015–2025).

As shown in Figure 3a, the annual distribution of publications has been gradually increasing over the last decade. Output was modest before 2019 but noticeably increased starting from 2020 onward with the coronavirus disease 2019 pandemic. The peak in 2024, with n = 9, reflects enhanced international focus on infectious disease prevention and the emerging role of digital health. Despite a slight decrease in 2025, with n = 7, the overall trend indicates continued scholarly interest.

Geographical mapping of contributions (Figure 3b) reveals Asia as the major source, particularly China (n = 10) and India (n = 4), followed by Europe, Africa, and North America. Interestingly, South Africa and Nigeria made major contributions to community-based approaches, whereas Europe contributed significantly to theoretical and methodological advancements. This pattern indicates that research on TB prevention reflects both regional disease burden and research capacity.

The journals in which TB prevention studies are published are shown in Supplementary Figure 1. Most studies were published in public health and multidisciplinary journals, with the largest proportions in International Journal of Environmental Research and Public Health, Frontiers in Public Health, and BMC Public Health. Additional studies were published in journals such as Journal of Health Communication and PLoS One, contributing to methodological diversity.

### Methodological approaches

**Figure 4:**
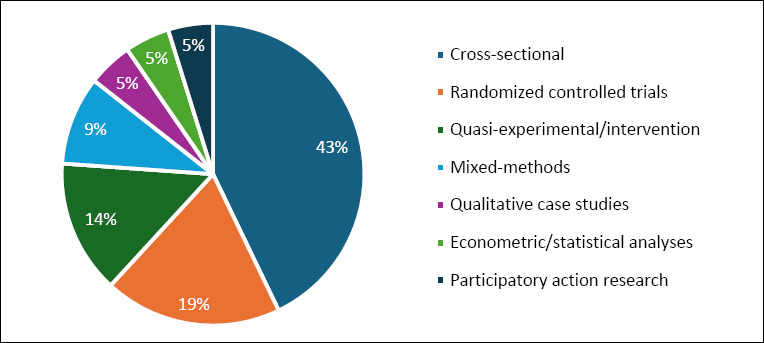
Methodological approaches in tuberculosis prevention studies (2015–2025).

The methodological distribution of the 49 included studies showed that cross-sectional designs were the most common (43%), followed by randomized controlled trials (19%), quasi-experimental or intervention studies (14%), and mixed-methods designs (9%). Furthermore, a limited number of studies applied qualitative case studies, econometric or statistical analyses, or participatory action research designs (each approximately 5%) (Figure 4). While quantitative approaches were most common, only a limited proportion of studies employed longitudinal or mixed-methods designs, limiting deeper insights into sustainability and causal mechanisms.

### Theory classification

**Figure 5:**
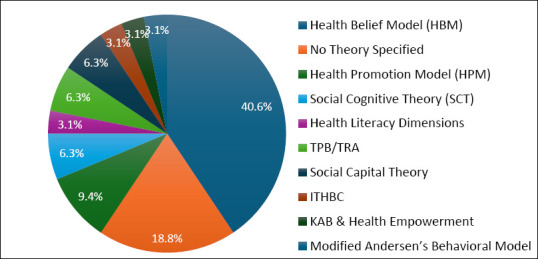
Theory classification of tuberculosis prevention studies (2015–2025).

Out of the 49 included studies, the HBM was the most frequently applied theoretical framework (40.6%), followed by studies without a specified theoretical framework (18.8%) and the HPM (9.4%). Other theories, including Social Cognitive Theory, theory of planned behavior or theory of reasoned action, and Social Capital Theory, were used less frequently (each approximately 6.3%). Additional frameworks, such as HL dimensions, Integrated Theory of Health Behavior Change, knowledge–attitude–behavior, and health empowerment, as well as the Modified Andersen’s Behavioral Model, were applied in a limited number of studies (approximately 3.1%). The distribution is presented in Figure 5.

This distribution highlights the dominance of HBM in TB prevention research but also indicates reliance on single-level models. Notably, most frameworks paid limited attention to structural and cultural determinants, which helps explain the persistent gap in integrating contextual and systemic factors. The proposed EHPCF addresses this limitation by reconceptualizing HL as a multidimensional competency within socioecological and digital contexts. A detailed breakdown of content categories is provided in Supplementary Figure 2.

### In-depth thematic analysis

Four broad themes were identified:

**(1) Importance of HL:** HL was consistently identified as a predictor of preventive behaviors, with studies using either standardized scales or non-standardized measures [6, 7, 17, 19]. These findings confirm the predictive validity of HL in TB prevention.

**(2) Community-based HP:** Peer education, school-based programs, and stigma-reduction workshops improved self-efficacy and health-seeking behaviors [8, 12, 13]. However, most interventions lacked longitudinal evidence of sustainability.

**(3) Digital health and media innovations:** Mobile health and electronic health interventions increased awareness and adherence [8, 9, 15, 30], but access inequities limited their overall impact [31]. Media literacy training also emerged as a promising yet underutilized strategy [13, 14].

**(4) Structural and cultural determinants:** Migration, poverty, stigma, and weak system responsiveness influenced TB outcomes [11, 16, 18, 32]. Few studies have integrated these determinants into formal models, thereby limiting their explanatory power.

### CIMO-based synthesis

Table 1 summarizes the key findings using the CIMO framework. Context included high TB burden populations, vulnerable groups, and digital divide settings [30, 32–34]. Intervention components included HL and HP programs, digital platforms, and community engagement strategies [15, 35–39]. Mechanisms included self-efficacy, stigma-reduction, trust, and social support [11, 13, 32, 38]. Outcomes included improved preventive behaviors, treatment adherence, and sustainability [9, 35, 40, 41]. Supplementary Table 2 provides detailed study characteristics.

**Table 1:** CIMO-based synthesis of tuberculosis prevention studies (2015–2025).

Context (C)	Intervention (I)	Mechanism (M)	Outcome (O)	References
High TB burden populations (urban poor, migrants, adolescents)	Community-based HL/HP programs (peer education, school-based training, outreach)	Increased self-efficacy; enhanced health-seeking orientation	Improved adoption of preventive behaviors (mask use, screening uptake)	[10, 41]
Settings with digital divides (rural, older adults, low-resource)	mHealth/eHealth literacy interventions, SMS reminders, telehealth	Improved access to information; reduced knowledge gaps	Greater TB awareness, medication adherence, early diagnosis	[8, 9, 15]
Communities with high stigma and cultural barriers	Stigma-reduction workshops, family engagement, social mobilization	Stigma-reduction; normalization of prevention; trust in providers	Increased service utilization; reduced delay in diagnosis	[13, 32, 42]
Low socioeconomic status, weak system responsiveness	Multi-sectoral collaboration, subsidies, policy interventions	Strengthened social support; enhanced system trust	Sustainable TB control programs; long-term adherence	[16, 17, 19]
Cross-cultural and educational contexts	HL training in curricula; cultural adaptation of tools	Improved critical awareness; transferability across settings	Broader HL improvements; culturally sensitive interventions	[6, 12]
High TB burden populations (urban poor, migrants, adolescents)	Community-based HL/HP programs (peer education, school-based training, outreach)	Increased self-efficacy; enhanced health-seeking orientation	Improved adoption of preventive behaviors (mask use, screening uptake)	[10, 41]
Settings with digital divides (rural, older adults, low-resource)	mHealth/eHealth literacy interventions, SMS reminders, telehealth	Improved access to information; reduced knowledge gaps	Greater TB awareness, medication adherence, early diagnosis	[8, 9, 15]
Communities with high stigma and cultural barriers	Stigma-reduction workshops, family engagement, social mobilization	Stigma-reduction; normalization of prevention; trust in providers	Increased service utilization; reduced delay in diagnosis	[13, 32, 42]
Low socioeconomic status, weak system responsiveness	Multi-sectoral collaboration, subsidies, policy interventions	Strengthened social support; enhanced system trust	Sustainable TB control programs; long-term adherence	[16, 17, 19]
Cross-cultural and educational contexts	HL training in curricula; cultural adaptation of tools	Improved critical awareness; transferability across settings	Broader HL improvements; culturally sensitive interventions	[6, 12]
High TB burden populations (urban poor, migrants, adolescents)	Community-based HL/HP programs (peer education, school-based training, outreach)	Increased self-efficacy; enhanced health-seeking orientation	Improved adoption of preventive behaviors (mask use, screening uptake)	[10, 41]

## DISCUSSION

This review synthesized 49 studies on TB prevention published between 2015 and 2025. The four dominant themes identified through analysis included HL as a determinant, community-based HP, digital health innovations, and structural and cultural determinants. These findings indicate that although research on TB prevention has increased, it remains fragmented, with limited integration of theoretical and contextual perspectives.

### HL as a core determinant

HL was consistently predictive of TB preventive practices, although measurement approaches varied among studies. Some studies used standardized tools such as the HLS-EU-Q [6], whereas others employed context-specific or ad hoc measures [10]. This variation limited comparability across studies. However, the positive predictive relationship between HL and preventive practices is consistent with previous findings, including studies conducted in the United Kingdom demonstrating that HL predicts health-related outcomes [6, 12, 43].

### Community-based HP

Community-based interventions, such as peer education, school programs, and stigma-reduction workshops, improved self-efficacy and health-seeking behaviors [32, 37, 38]. Similar findings were reported in Nigeria and Turkey, where HBM-based interventions increased knowledge and preventive practices [10, 11]. However, most interventions were time-bound and lacked evidence of long-term sustainability, consistent with findings from meta-analyses conducted in Ethiopia [18].

### Digital health and media innovations

The development and application of mobile health and electronic health platforms represent a paradigm shift in TB prevention strategies. Short-message service (SMS) messaging and mobile applications have increased awareness and adherence among individuals [35, 36]. Previous studies have demonstrated that digital media improve HL and promote preventive behaviors [8, 44]. However, disparities in access remain a major challenge, particularly in rural settings [18, 30]. In addition to digital literacy, media literacy training is emerging as a strategy to improve critical evaluation of health information [13, 14].

### Structural and cultural determinants

Socioeconomic status, migration, stigma, and weak health system responsiveness significantly influenced TB prevention outcomes. Studies conducted in South Africa and India demonstrated that stigma and poverty contributed to delays in diagnosis and treatment. Similarly, Makgopa *et al*. [32] reported that stigma reduced quality of life and increased non-compliance. Despite this evidence, most studies treated these determinants as background variables rather than integrating them into explanatory models. This gap reflects broader limitations in the literature on social determinants of health and highlights the need for systematic integration of these factors into intervention design [17].

To further contextualize these findings, insights from the author’s ongoing mixed-methods dissertation in Surakarta provide additional interpretive support. Qualitative findings indicate that TB prevention behavior is shaped by sociocultural contexts that influence how individuals interpret health information, perceive risk, and engage in preventive practices. Social norms, stigma, and community traditions influence willingness to seek care and adherence to preventive behaviors. In addition, social support from family and community, along with observational learning, plays a key role in reinforcing preventive practices in daily life. These findings highlight the importance of integrating HP and HL within culturally responsive approaches.

At the institutional level, healthcare providers, health facilities, and community health volunteers influence patient understanding and behavior through health education, interpersonal communication, and ongoing support. These processes enhance self-efficacy and motivation to maintain preventive practices, consistent with key constructs of HP models such as EHPCF, which emphasizes interactions among individual, social, and institutional factors. These insights complement the interpretation of the review findings and do not constitute part of the systematic review dataset.

### Contribution of EHPCF and CIMO

The novelty of this review lies in two main contributions. First, EHPCF reconceptualizes HL as a multidimensional competency encompassing personal, media, sociocultural, systemic, and environmental domains. Unlike previous reviews that primarily described determinants [19, 33, 45], this framework provides a structural model linking HL to socioecological systems and digital determinants.

Second, the application of CIMO provides an explanatory perspective on how interventions operate across contexts. It enables the identification of mechanisms through which interventions produce outcomes. For example, stigma-reduction workshops in high-burden populations activate mechanisms such as trust and social support, leading to increased service utilization [13, 32, 42]. Only 32% of studies incorporated structural determinants into models; EHPCF addresses this limitation by embedding these determinants within systemic competencies.

Importantly, EHPCF is derived from the synthesis of included studies and represents a conceptual output of this review. It is further used as a guiding model in the author’s ongoing mixed-methods research to examine TB prevention behavior within integrated HL and HP perspectives.

### Implications

The findings have important theoretical and practical implications. Theoretically, they support the development of integrative frameworks that combine HL, HP, digital health, and structural determinants. In practice, TB prevention programs should adopt multi-component strategies that include HL training, stigma reduction, and digital health integration.

These findings align with global TB control priorities and support efforts to achieve the End TB targets and Sustainable Development Goals 2030 [1, 40]. Qualitative insights further emphasize the importance of sociocultural context, community engagement, and social support in shaping preventive behavior. Therefore, effective TB prevention strategies must extend beyond individual-level interventions to include community-based and culturally responsive approaches.

### Limitations

This review has several limitations. The inclusion of only Scopus-indexed journals introduces potential publication bias. The predominance of cross-sectional study designs limits causal inference. The use of heterogeneous measurement tools prevented meta-analysis. In addition, restricting the search to English-language publications may have introduced language bias.

### Future research

Future research should prioritize longitudinal and mixed-methods designs to evaluate sustainability and causal mechanisms. Further studies are needed to validate EHPCF across diverse settings and to address digital inequities. The use of explanatory frameworks such as CIMO can enhance the development of context-sensitive and action-oriented TB prevention strategies.

Future interventions should assess competency development across all EHPCF domains to predict long-term sustainability. Formal validation and application of EHPCF across diverse cultural contexts will enhance its utility in guiding TB prevention programs globally.

## CONCLUSION

This systematic review, synthesizing 49 studies, provides a comprehensive understanding of TB prevention by integrating HL, HP, digital health, and socio-structural determinants within a unified analytical framework. The results demonstrate that HL consistently functions as a strong predictor of preventive behaviors, while community-based HP interventions enhance self-efficacy and health-seeking practices. Digital health innovations, including mobile health and electronic health platforms, contribute to improved awareness and adherence; however, their effectiveness is constrained by inequitable access and digital divides. Importantly, structural and cultural determinants such as stigma, poverty, and migration significantly influence TB prevention outcomes, yet they remain insufficiently incorporated into existing intervention models. The application of EHPCF and CIMO provides an integrated and explanatory framework, highlighting that context-specific mechanisms, including trust, social support, and stigma-reduction, play a critical role in translating interventions into measurable outcomes.

A key strength of this review lies in its integrative and theory-driven approach, combining EHPCF and CIMO to move beyond descriptive synthesis toward a mechanistic understanding of TB prevention strategies. The inclusion of diverse study designs and global contexts enhances the comprehensiveness of the findings, while the structured synthesis allows identification of consistent patterns across heterogeneous evidence. Additionally, the review contributes conceptually by positioning HL as a multidimensional competency embedded within sociocultural, systemic, and digital contexts, thereby advancing existing theoretical models.

In conclusion, this review underscores that effective TB prevention requires a holistic, ecosystemic approach that integrates HL, HP, digital health, and structural determinants, rather than addressing them in isolation. The combined application of EHPCF and CIMO offers a robust framework for designing context-sensitive, sustainable, and scalable interventions. These findings provide a strong foundation for future research and policy development to strengthen TB prevention efforts, particularly in high-burden, resource-constrained settings.

## DATA AVAILABILITY

The supplementary data can be available from the corresponding author upon request.

## AUTHORS’ CONTRIBUTIONS

SS: Conceptualization. SS and ESS: Methodology. SS and SA: Software. SS, ESS, SA, and AL: Validation, formal analysis, data curation, visualization, and drafted and edited the manuscript. SS, SA, and AL: Investigation. All authors have read and approved the final version of the manuscript.
